# Technostress Dark Side of Technology in the Workplace: A Scientometric Analysis

**DOI:** 10.3390/ijerph17218013

**Published:** 2020-10-30

**Authors:** Giorgia Bondanini, Gabriele Giorgi, Antonio Ariza-Montes, Alejandro Vega-Muñoz, Paola Andreucci-Annunziata

**Affiliations:** 1 Department of Human Science, Università Europea di Roma, 00163 Roma, Italy; giorgia.bondanini@gmail.com (G.B.); gabriele.giorgi@unier.it (G.G.); 2 Social Matters Research Group, Universidad Loyola Andalucía, 14004 Córdoba, Spain; ariza@uloyola.es; 3 Faculty of Business Administration, Universidad Autónoma de Chile, Santiago 7500912, Chile; 4 Faculty of Education, Pontificia Universidad Católica de Chile, Santiago 7820436, Chile; pmandreu@uc.cl or; 5 Education Research Center, Universidad Bernardo O’Higgins, Santiago 8370993, Chile

**Keywords:** mental health, technostress, information-technology, dark side, work, information overload, scientometrics

## Abstract

This article aims to provide a critical review of the scientific research on technostress. As such, global references in this field are identified and highlighted in order to manage pre-existing knowledge and establish future ‘bridges’ among researchers, and to enhance the presently dispersed understanding of this subject. A scientometric meta-analysis of publications on technostress was conducted to achieve this objective. Mainstream journals from the Web of Science (WoS) were used to identify current topics, relevant journals, prolific authors, institutions, and countries, ‘schools of thought’ and the thematic areas around which current technostress debate revolves. In this article a significant contribution comes from the use of the scientific activity itself, together with scientometric meta-analysis techniques and the application of this scientific activity, its impact and relational character, to discover relevant countries, research organizations and authors which can constitute a global reference to demarcate this knowledge frontier, and who lead the ‘critical mass’ of global technostress researchers. This study also distinguishes between the relevant themes studied, with co-keywords plus bibliographic coupling citation, and examines the kind of stress the most prolific authors have considered and, therefore, to discover those topics which should be studied further to deepen this research field, in search of a post-disciplinary knowledge that allows unity of focus in technology and psychology.

## 1. Introduction

The concept of stress in the scientific field was officially born in the 1940s in the theory of Walter Cannon [1] who adopts it and defines it in association with the concept of “homeostasis”. Furthermore, he illustrates the so-called attack and flight response, consisting of physiological modifications in which a central role is played by the sympathetic nervous system, which brings the individual into a state of reaction, activation and reactivity [2]. However, the first demonstration of connection between stress and disease comes later. [3].

It is possible to consider Selye’s theory in the context of technostress? While tremendous advances in the field of information and communication technology (ICT) have resulted in significant benefits for human society, growing evidence shows the ‘‘dark side’’ of ICT for individual users and organizations [4,5]. Technostress (TS), defined as ‘‘stress experienced by end users in organizations as a result of their use of ICT’’ [6] (pp. 417–418), is one major ‘‘dark side’’ of ICT.

It has been noted that the dramatic proliferation level of mobile devices and wireless communication technology use has significantly impacted on the growth of mobile commerce in recent years, considered as a value-added service and source of revenue generation [7,8,9]. With the increasing use of smartphones, mobile apps are deemed to be an integral part in the daily life of users, who use them an average of 30 h per month, according to New Google’s and Ipsos’s research. Shopping via app is becoming popular globally. The latest United Parcel Service (UPS) of America survey [10] found that four out of five interviewees used a brand app for shopping. The importance of apps has been recognized by both consumers and marketers [11].

While there have been several attempts in organizations to counteract techno-stressors [12,13], a scientific approach proven to be highly effective in producing positive change is Positive Psychology [14,15], with its derivative Positive Technology [16,17,18]. In Positive Psychology personal experiences can be leveraged to foster well-being and personal growth. Similarly, Positive Technology is used for improving the quality of our personal experience which occurs at three different domains of technology use/design: hedonic (generate positive experiences), eudemonic (support individuals in reaching “engaging and self-actualizing experiences”), and social/interpersonal (improve connectedness between individuals or groups) [17,19].

Despite the many areas of interest, the number of scientific papers addressing technostress does not cover these areas, especially compared with the volume of articles published on stress and technostress in general. This article aims to provide a critical review of the scientific research on technostress, a concept that has emerged based on multiple streams of thinking. As such, global references in this field are identified and highlighted to manage pre-existing knowledge in order to establish future ‘bridges’ among researchers and to enhance the presently dispersed understanding of this subject. A scientometric meta-analysis of publications on technostress is conducted to achieve this objective. Mainstream journals from the Web of Science (WoS) are used to identify current topics, most relevant journals, most prolific authors, institutions, countries, ‘visible’ and ‘invisible’ collaborative colleges and the thematic areas around which current technostress debate revolves.

A significant contribution comes from the use of the scientific activity itself, together with scientometric meta-analysis techniques and the application of this scientific activity, its impact and relational character, to discover relevant countries, research organizations and authors which can constitute a global reference to demarcate this knowledge frontier, and who lead the ‘critical mass’ of global technostress researchers. This study also distinguishes between the relevant themes studied, and, therefore, aims to discover those topics which should be studied further to deepen this research field.

Section 2 reviews the related literature to accomplish the main objective. Section 3 presents the research methodology, and Section 4 comments on the main results obtained. Section 5 critically discusses these results. The conclusion and the main limitations are presented in Section 6 and Section 7, and the list of bibliographic references used in this study are listed in the references section.

## 2. Background

To contextualize the scientometric meta-analysis of the published academic literature that will be carried out later, it is necessary to provide a critical and updated vision of what is understood by techno-stress, as well as its main focuses of attention in the work environment. Technostress is commonly defined as a modern disease of adaptation caused by an inability to cope with new computer technologies, affecting mental health in a manner which may manifest as a struggle to accept computer technology, or as over-identification with computer technology [6,20,21]. It is also defined more generally as any negative impact on attitudes, thoughts, behaviors, or body physiology that is caused either directly or indirectly by technology [22]. Research on technostress tends to focus on business users of technology, and particularly the mandatory use of technology.

Brod [21], one of the pioneering authors to study technostress, notes that this phenomenon is based on the root concept of stress, including: (1) the internal state of the organism (or strain); (2) an external event (or stressor); and (3) an experience that arises from an ongoing transaction between a person and the environment [23]. This is further derived from general stress in the workplace, which is considered as the harmful physical and emotional responses that occur when job requirements do not match the worker’s capabilities, resources, and needs [24]. In this sense, technostress is commonly described in association with an individual’s role in the workplace and the tasks the individual is assigned to perform with technology as part of that role [22]. Specifically, technostress occurs when an individual has a negative evaluation of their experience when carrying out tasks using technology at work and is distinct from studies of general work stress [25,26]. Brod [21] also defined technostress as ineffective coping with technology that results in distress. Use of information and communications technologies (ICT), such as cell phones, voice mail, e-mail, and instant messaging, can challenge employees by creating a range of stressors, including overload, role ambiguity, and job insecurity [27,28].

Ragu-Nathan et al. [6] used a transactional approach to describe a combination of tasks and roles that create stress (e.g., increased information, processing requirements, role ambiguity) and situational factors, such as organizational mechanisms, that buffer the impact of ICT (e.g., perceived control) [6]. Technostress affects job satisfaction, organizational commitment, and employee outcomes (e.g., absenteeism, turnover). It is defined using two dimensions: technostress creators and technostress inhibitors [29]. On the one hand, technostress creators include techno-invasions (constant connectivity that invades life), techno-overload (simultaneous, different streams of information that increase the pace and volume of work), techno-uncertainty (from a change or upgrade to hardware, software, or applications; ambiguity around expectations related to changes), techno-insecurity (employees feel threatened by job loss to technology or to other people with more ICT understanding), and techno-complexity (the inherent complexity of ICT that users find difficult to understand, which leads to feelings of incompetence). On the other hand, technostress inhibitors include literacy facilitators (knowledge sharing, teamwork, end-user training, user guides), technical support provision (assistance provided to employees to reduce techno-complexity and techno-uncertainty), and involvement facilitation (mechanisms to engage employees in adopting systems).

More recently, Salanova et al. [30] consider technostress as a negative psychological state related to current or future use (or abuse) of technology. This overarching concept encompasses two experiences: techno-addiction and techno-strain. Studies of techno-addiction are based on workaholism research with compulsive use and excess devotion of time associated. It has an uncontrollable “have to” pressure paired with anxiety when not using ICT. Techno-strain includes four interrelated constructs —anxiety, fatigue, skepticism, and inefficacy—that occur in a chain reaction relationship. Anxiety is an overactivated emotional response of fear, apprehension, or agitation characterized by high physiological activity and tension. Fatigue is an affective response typified by low activation from information overload. Skepticism is attitudinal and characterized by cynicism, mirroring burnout. Users feel exhausted or discouraged and display distant, detached, and indifferent attitudes toward technology. Finally, the cognitive dimension of inefficacy involves perceived levels of ICT efficacy. According to Salanova et al. [30], with chronic anxiety, fatigue, and skepticism, users’ ICT self-efficacy lowers. The technology demands predictor refers to the psychological or physiological cost of the sustained effort required of employees related to ICT use in the organization. Such demands can include work overload (e.g., excess work, attentional demands), ergonomic stresses, the pace of work (i.e., time to perform tasks is less than available time), role ambiguity (tasks associated with ICT are poorly defined), and monotonous and unchallenging ICT tasks. At the societal level, formation of human relationships around use of ICT can create social isolation, emotional overload, or role conflict (e.g., multiple virtual teams operating differently, old, and new systems operating concurrently). At the organizational level, demands may relate to competitive advantage in the labor market. This can take the form of job insecurity (i.e., jobs at risk because of ICT), organizational culture (e.g., limited ICT choices), or work–life conflicts [30].

Therefore, the consequences of technostress are physiological, psychosocial, organizational, and societal. Physically, workers can develop problems or health can deteriorate. Psychosocial problems (e.g., anxiety, job dissatisfaction, decreased work engagement) can lead to mental exhaustion or user self-belief of incompetence. Employers can observe low performance and absenteeism, excess personal use of ICT, or low commitment and retention. In the organizational context, ICT abuse can cause social contact and networks to deteriorate or can create financial problems [30]. Finally, at the societal level, some authors such as Martínez-Córcoles et al. [31] highlight that technology could be a threat to our established set of norms and patterns of behaviour which make us adaptive in our environment, and therefore brings negative emotional reactions, anxiety and fear. This ambivalence is expressed by technophobia (rejection and/or avoidance of technology) and technophilia (attraction and enthusiastic adoption of technology).

So far, scientific publications on technostress have focused on two major areas: the social media-technostress relationship, and the link between technostress, work environment and quality of life.

### 2.1. Technostress and the Use of Social Media in the Workplace

With the rapid development of mobile technology and smart devices, social media such as wikis, blogs, instant messaging (IM), and social networking sites (SNSs) have penetrated people’s daily life. These tools can be used for socializing, entertainment, self-promotion, communication, and information seeking [32] by almost anyone, anywhere, at any time. Attracted by their prevalence and convenience, social media are now becoming an indispensable part of organizational life [33].

Individuals may bring personal activities into the workplace, resulting in life-work conflict and eventually leading to exhaustion [34]. Furthermore, the widespread use of social media in the workplace might lead to loss in employees’ productivity as a result of time wasting and distraction at work [35]. For example, a study by Nucleus Research found that full access to Facebook at work results in a 1.5 percent drop in productivity [36]. Likewise, as noted by Brooks [37] and Bucher et al. [38], individuals who depend on social media excessively are likely to suffer feelings of conflict, overload, and lower well-being. These feelings may ultimately increase technostress caused by the usage of social media, and thereby result in decreased job performance [37]. Therefore, the phenomenon of excessive use of social media in the workplace has become a significant problem for organizations and deserves more attention from scholars [39].

One detrimental unintended consequence of using social media at work has been identified as the stress induced by social media, or social media-induced technostress [38]. This stress could arise for several reasons. For example, employees may become overloaded by accessing and mentally processing information related to both work and personal life during work hours [20,38]. Similarly, checking social media at work for personal reasons may blur the line between work and home life and lead to the invasion of personal life into the workplace [6,22].

A study by Brooks et al. [40] draws on Person-Environment Fit to investigate the relationship between social media-induced technostress and job performance in IT professionals, and the moderating effect of job characteristics on this relationship. The sample used for analysis (N = 750) consisted of individuals spanning 42 different IT job titles. This investigation underlined that social media-induced technostress associated with using personal social media at work had a direct negative effect on job performance. Moreover, high levels of three of the job characteristics significantly reduced this negative relationship, with one characteristic, task significance, marginally reducing this effect.

### 2.2. Technostress, Work Environment and Quality of Life

Over the past decade, the workplace has experienced significant changes as a result of Information and Communication Technologies (ICTs) and subsequent digital transformation [41]. Such technological, cultural, and organizational changes have redefined business models and competition. As evidenced by the shift from the Enterprise 1.0 to the Enterprise 2.0 business models, ICTs offer companies increased productivity and efficiency [42]. At the same time, introduction of ICTs can pose a threat to both a company and its employees through misuse, abuse, and overuse, resulting in technostress [43]. This emerging risk seems to have become more evident in the past ten years, as a consequence of the 2008 economic crisis. This difficult and challenging economic context was demonstrated to have negatively impacted workers’ mental health in any case, due to workers’ perception of the crisis, lack of social support, and increased job stress [44,45].

At a time when computer science, robotics and artificial intelligence occupy a greater quantity of work space, even going so far as replacing it completely in specific tasks (job killing), it seems useful to try to understand what the related problems and novel perspectives [46]. Drawing inspiration from Ritter [47], some ideas related to new technologies can be proposed: (1) they substantially change expectations in relation to conditions and content of work, and the need to increase freedom of development and personal integration; (2) they facilitate the creation of critical knowledge in new sections of the population, concerning economic development and traditional concepts of responsibility, authority and value; (3) they induce new ways of analyzing systems of attitudes and personal values, due to the complexities and articulations they imply; (4) they induce an increase in the attention paid to levels of personal safety, privacy and quality of life, levels that must be absolutely respected and guaranteed; and (5) they have crucial effects in the planning of work.

Furthermore, supported by the technological advancement of the Internet, which people can access at any time and from most locations, Social Network Services (SNSs) have penetrated our daily lives [48]. The positive aspects of SNSs, such as social support, perceived usefulness, and perceived enjoyment, have been found to increase user satisfaction [49]. Individuals expect the use of SNSs to result in improvements in their relationships and productivity with respect to communication and technology. Accordingly, users invest a considerable amount of time in SNSs. However, recent research has shown that users become depressed [50], and their productivity at work is affected due to increases in the time spent using SNSs. New communication technologies, including SNSs, offer benefits but also create new problems and ultimately lead to a dilemma regarding the extent of technology usage [51].

### 2.3. Other Topics

Other topics of interest regarding technostress can be consulted in the literature review carried out by Mahapatra and Radhakrishna [52]. These authors identify a series of general studies on technostress that emphasize different variables such as work exhaustion [43], the big five personality traits [53], performance [54] and work life conflict [55]. Other research focuses on a specific technology use: e.g., telemedicine technologies [56] or information security [57]. Finally, Mahapatra and Radhakrishna [52] identify another group of investigations that focus their attention on different working professionals, such as administrative staff [58], sales professionals [59] or librarians [60].

Technostress is an emerging theoretical construct that has developed over less than two decades and has emerged from multiple streams of thinking, which do not allow a clear vision of its scope, references, and relevance.

## 3. Methods

This research uses Scientometry as a systemic working method. According to Vega and Salinas [61], the main objective of this methodology is to assess scientific evolution and development, as well as to judge scientific policies in relation to certain aspects of economics and society. From this point of view, the scientometric meta-analysis presented here focuses on technostress studies, taking Web of Science (WoS) articles as a reference, given its recognized quality among researchers worldwide [62]. As a search vector [63], the “technostress” construct present in articles in the Journal Citation Report (JCR) indexes, both SCI-EXPANDED (170 disciplines) and SSCI (50 social science disciplines), for a data recovery period between 1975 and 2019, is considered thematically. Following the recommendations of Archuby et al. [64] the following search vector was used: (TS = (Technostress)) AND TYPES OF DOCUMENTS: (Article), Indexes = SCI-EXPANDED, SSCI Time period = 1975¨C2019. Once these records are obtained from 67 metadata fields extraction (grouped as: author identification, localization and affiliation; article/source identification, access, recuperation codes and citation; keywords, abstract, cited references, and funding), it will be analyzed, using bibliometric rigor, whether increases in the scientific production under study achieve a critical research mass, in an exponential growth form [65,66,67]. Then, a determination of contemporary literature follows, determined by the time period of articles produced [65,68]. Table 1 identifies the inputs and outputs of each of these analytical methods [69].

Next, quantity (production), quality (impact) and relationship [61,70], prolific authors’ concentrations according to Lotka’s Law [71,72], nucleus journals according to Bradford’s Law [73,74,75,76,77,78], and highly cited articles according to the Hirsch index [79,80,81,82,83,84] will be analyzed by scientometry. In another phase, through the VOSviewer [85,86], analysis by co-authors at the level of affiliation with country/region, affiliation with institutions and affiliation with other authors will take place. As well as thematic study, high-use ‘keywords plus’ (KWP, keywords corrected by WoS) according to Zipf’s Law [87,88], common bibliographic references with bibliographic coupling [89] and classic authors on stress research, have also been used as references on technostress research.

## 4. Results

**Figure 1:**
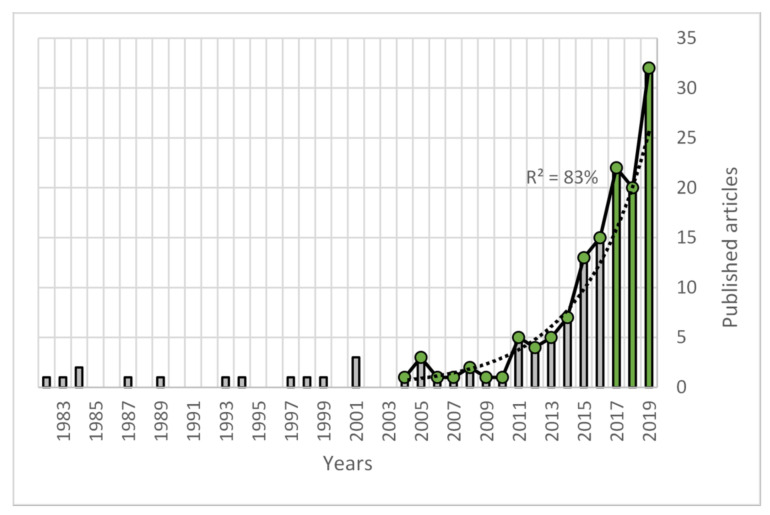
World scientific production growth.

As a first result, we must highlight the exponential growth achieved by world scientific production on technostress since 2003 (see Figure 1), observing a critical mass that allows us to prospect a future research growth on this topic and to estimate a replacement that allows us to generate contemporary knowledge. Figure 1 details an adjustment with an R^2^ of 83% and a contemporary knowledge half-period (represented in green bars) between 2017 and 2019. These data show that in recent years “critical mass” has been consolidating around research into technostress.

### 4.1. Concentration Analysis

Figure 1 gives us a first concentration account, regarding contemporary knowledge concentrated in the last three years. In the period analyzed, a total of 147 articles have been published in which 296 authors have participated. To determine the list of the most productive authors, Price’s Law [66] and López’s recommendations [90] were used. In this way, the square root of the total number of authors (296) was calculated, obtaining a value of 17. These are the most prolific authors, all with 3 or more publications on technostress. Given that the authors who occupy positions 18 and 19 had also published three articles on the subject, it was considered appropriate to include them in the prolific authors group, as can be seen in Table 2. Professor Monideepa Tarafdar from the University of Lancaster stands out with the publication of 12 articles on technostress in this period. The contribution of the other most productive authors ranges between three and five articles per author.

Regarding the institutional affiliation of these authors, Table 2 shows that five of them belong to the University of Science and Technology of China and another three work at the Otto-Friedrich-Universität Bamberg of Germany.

Additionally, Table 2 shows that this authors group in their doctoral training specializes in in Management and their publications are mainly focused on Information Science & Library Science, Computer Science and Psychology journals.

To continue with the literature concentration analysis, and following the Kumar recommendations [77], the Bradford zones were determined (the estimation procedure can be found in Appendix A). This methodology distributes the 147 articles under analysis among a total of 82 journals (see Table 3). However, this distribution presents a high concentration degree around the seven journals that form the core in Zone 1, since 35% of the articles published on technostress are concentrated in only 9% of journals (see Table 4). These journals lead the current debate on the causes and consequences that the use of technology generates. As can be seen in Table 4, according to WoS (2018), five belong to the first quartile (Computers in Human Behavior, Journal of Management Information Systems, Information Systems Journal, Journal of The Association for Information Systems and Telematics and Informatics), while the remaining two are located in the third quartile (Behavior Information Technology and Information Technology People). Most of these journals fall into two Wos categories (“Information Science & Library Science” or “Computer Science, Information Systems”), although there are also journals that belong to the psychology and management areas.

**Figure 2:**
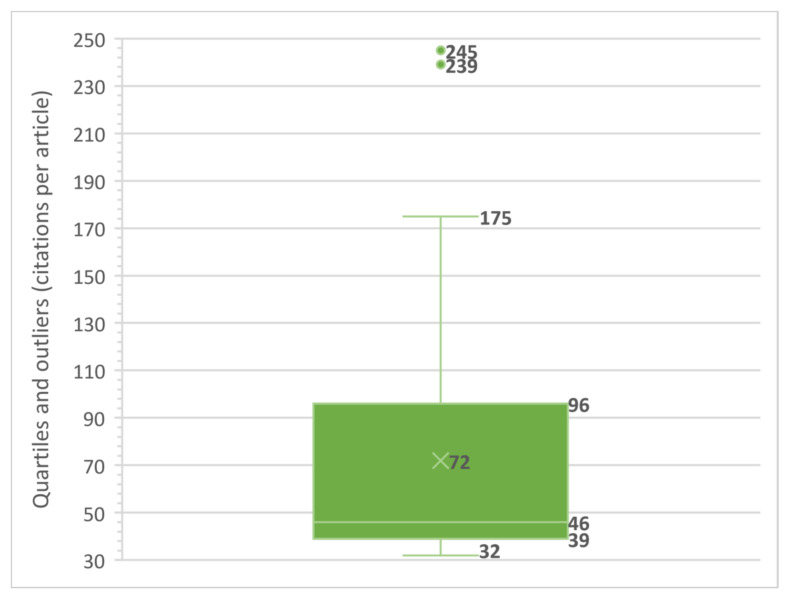
Box plot citations per h-index articles.

A final indicator of concentration or dispersion of academic research into technostress is associated with citations that this article set have received, presenting an h-index of 31 (31 articles cited 31 or more times); thus more than 20% (31/147) of the articles are considered as highly cited, with publication dates between 1982 and 2017. This set is described in Figure 2, presenting an average of 71 citations, values strongly biased towards the upper quartiles (Q2 of 46 to less than 96 citations and Q1 from 96 to 175 citations), and with two articles that, for this topic and period under study, have an atypical volume of citations. Thus standing out with 245 citations is the article ‘The dark side of smartphone usage: Psychological traits, compulsive behavior and technostress’ by Lee et al., published in 2014 in the journal ‘Computers in Human Behavior’ [91], and with 239 citations the article ‘Technostress: Technological antecedents and implications’ by Ayyagari et al., published in 2011 in the journal ‘MIS Quarterly’ [20].

### 4.2. Co-Authorship Analysis

The co-authorship analysis at three different levels (affiliation with country/region, affiliation with institutions and affiliation with authors) aims to distinguish a cohesive structure to show authors that, in a concerted way, have managed to network a critical knowledge mass in the field of study. The aggregation levels of institution and country/region allows us to show the power of this knowledge power and potential use regarding geopolitical affairs [92,93,94].

**Figure 3:**
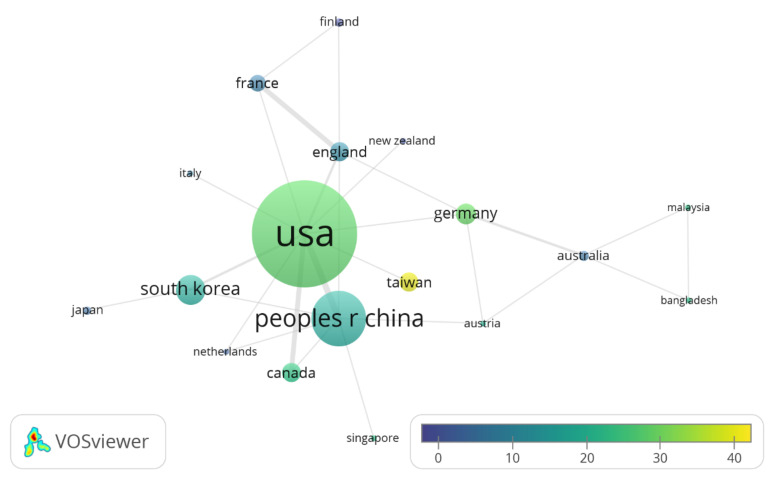
Country level co-authorship graph.

With regard to co-authors affiliations with countries, Figure 3 shows the national interaction consistently represented by the graph generated by VOSviewer, that occurs in author affiliation terms with eighteen countries among the articles under study, showing levels according to node size. Production varies from the USA with 50 articles to countries such as Bangladesh and Malaysia with only one document, and from a high average citations number received per article such as that of Taiwan in yellow (38.11) to that of Finland in violet (1.75). Although the United States in light green does not show the highest number of average citations, it presents a great centrality within the group given its national co-authorship with 10 other countries out of 18 (connected by direct lines, the width of the lines with Canada and the People’s Republic of China being a sign of greater collaboration), implying a position of knowledge power that cannot be equaled by any other nation.

**Figure 4:**
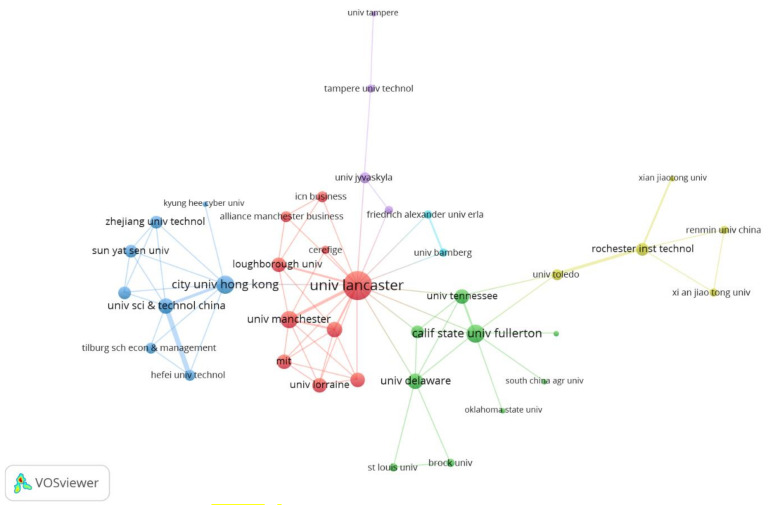
Organization co-authorship graph.

Regarding co-authors affiliation with institutions, Figure 4 consistently presents 38 of 198 institutions that interact in their scientific research at the organization level, highlighting the University of Lancaster’s centrality, becoming the nucleus that articulates the world production of knowledge in Technostress, as well as the bridge role exercised by the City University of Hong Kong (in light blue, Hong Kong, China), University of Toledo (in yellow, Toledo, OH, USA) and University of Jyväskylä (in violet, Jyväskylä, Finland), marking a boundary between a highly cohesive center of scientific production and a research periphery, to which these give access respectively to 7, 4, and 2 affiliation institutions.

**Figure 5:**
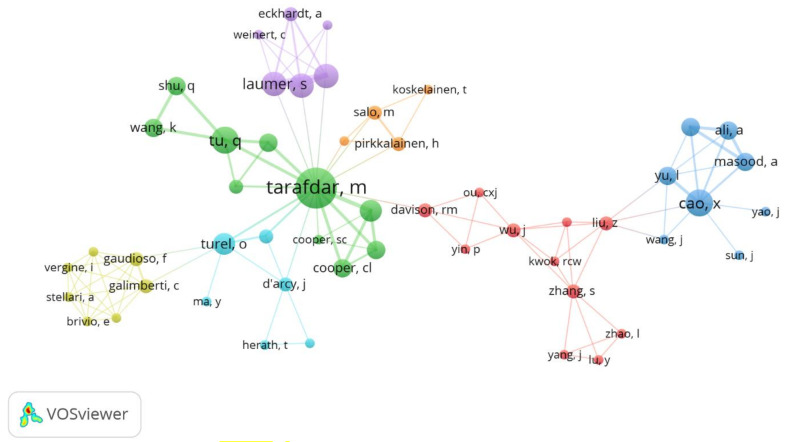
Researchers co-authorship graph.

Finally, concerning the interaction between authors, Figure 5 shows how the interaction of 52 researchers is generated, grouped into seven clusters, which are represented with different colors (yellow, light blue, green, violet, orange, red, and blue) that identify socially cohesive sets of authors that jointly produce knowledge. It is not exactly a small world with a single cluster where everyone collaborates with everyone [95], but it is possible to identify Tarafdar in green with a high production (largest node) and centrality (greater number of direct connections), consistent with the place that its affiliation occupies, the University of Lancaster in Figure 4. Also observed in the peripheral blue cluster are Cao and other authors from China, in the violet cluster the group of prolific authors affiliated with German universities (Erlangen-Nürnberg and Bamberg), and Turel of California State University - Fullerton in the light blue cluster, in direct connection with Tarafdar and serving as a bridge for the yellow cluster. Cao and the group from China seem to be opting for structural equivalence [96] within the network of prolific authors, especially due to the concentration of their work in journals listed in Psychology and the absence of direct connection with Tarafdar, who turns out to be the hegemonic author in this period.

### 4.3. Thematic Analysis

Once the concentration and degree of academic research on technostress has been analyzed, as well as the relational networks between authors, institutions, and countries, in this section we proceed to analyze the main research fronts that are being addressed.

**Figure 6:**
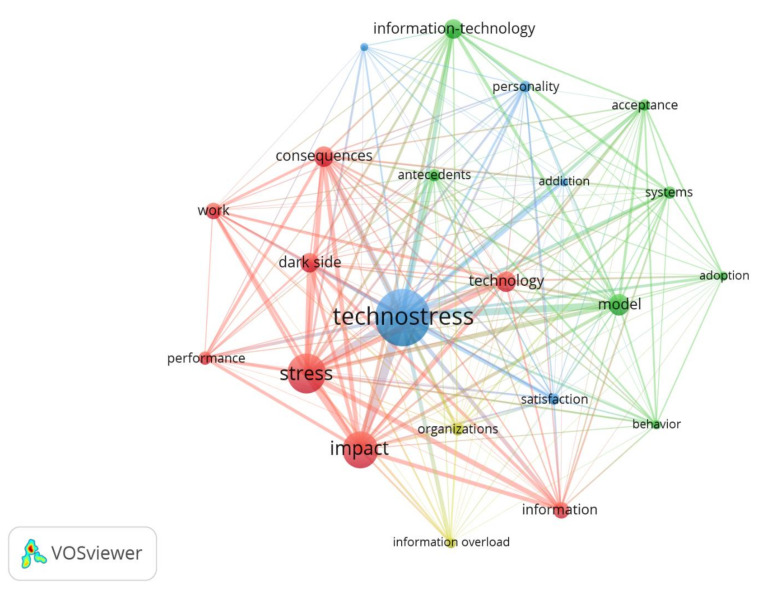
Co-words graph based on keywords plus (KWP).

In the thematic associations field, it is possible to study how research fronts are constituted based on the keywords. In this regard, the “keywords plus” (KWP) assigned by the Web of Science itself have been considered, given its settlement degree by its regular use in various scientific articles. These conceptual associations account for certain thematic variants with respect to a research topic. In Figure 6 four large areas of study can be seen, such as the KWPs dataset in red (stress, impact, consequences, dark side), green (information-technology, model, antecedents, acceptance), blue (technostress, personality, addiction, satisfaction) and yellow (organizations, and information overload), where ‘technostress’ in a blue node represents the greatest number of appearances (largest node), in a very dense network that tends to connect all the KWP (direct lines between nodes in the graph), based on their simultaneous use for the authors of various articles.

**Figure 7:**
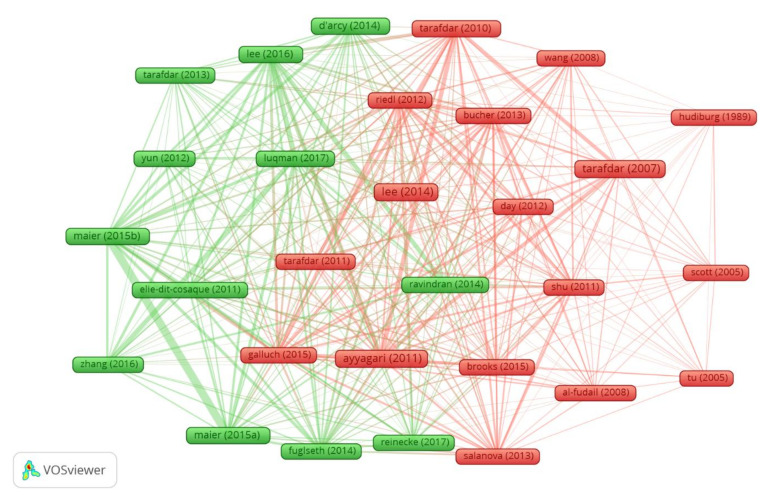
Bibliographic coupling graph.

The above can be deepened based on common bibliography use, as a referential thematic source used by the authors in their articles. This allows us to visualize a ‘school of thought’ presence developed in one or a set of institutions, either by simple co-authorship or the willful use of certain references. However, it also allows highlights an ‘invisible college’ existence, that is, authors who, without prior interaction, decide to substantiate their work on certain other authors, thus revealing a certain thematic/ideological approach to their articles. In this way Figure 7 shows an article set consistently connected by VOSviewer software (Centre for Science and Technology Studies - Leiden University, Leiden, The Netherlands) [85], which shares common references, segmenting the 31 articles identified by the h-index in two clusters (see Table 5 and a more detailed extension in Table A1) marked according to the colors green or red. Two articles are bibliographically coupled if their reference list shares one or more of the same cited documents, and the coupling strength (line width in the graph) increases as with the number of citations shared. In this way the coupling-citation tends to imply a reference structure for a specific research line. 

As can be seen in Table 5, with the exception of the texts of Brod [97], and Arnetz and Wiholm [98], the other 29 articles (within the h-index 31), are located, given their bibliographic coupling strength, in cluster 1 (red nodes) or in the cluster 2 (green nodes), implying that they are part of a references structure that thematically allows the estimation of choice between specific research lines.

Finally, Table 6 (see more detailed information in Table A2) below shows the classic authors ‘presence in stress research, who are also present in the articles on technostress meta-analyzed, managing to highlight the high presence of references to Lazarus and Cooper in the studies analyzed.

## 5. Discussion

This paper develops a scientometric piece of research at three different levels (concentration, co-authorship and thematic analysis) that try to offer a broad view of the scientific examination of technostress. We attempted to provide a rigorous analysis of the knowledge produced by scientific scholars of technostress. Technostress research has grown exponentially in the last years. Using scientometric methodology, we detected the main references in this knowledge area and “bridges” with related geographical, disciplinary and/or academic terms.

First, at the concentration analysis level, this article examined 147 articles published in 82 WoS journals. The results of this study showed a significant increase in the number of articles published in the last three years (2017–2019), which shows and confirms that it is a very topical issue. Applying Bradford’s law, scientometric analysis has identified seven journals that concentrate the debate and academic discussion about technostress, although one of these stands out over the others (Computers in Human Behavior, containing 16% of academic pieces). The other six journals amassed at most 4% of the total (Journal of Management Information Systems) or even less: Information Systems Journal (3%), Journal of The Association for Information Systems (3%), Behaviour Information Technology (3%), Information Technology People (3%) and Telematics and Informatics (3%).

These journals in which the academic debate about techno-stress is concentrated are located fundamentally in two scientific knowledge areas, one with a more technological nature (assigned to two WoS categories: Information Science & Library Science, and Computer Science, Information Systems) and another with a more psychological approach (assigned to the WoS categories: Multidisciplinary Psychology and Experimental Psychology). That most publications are from two approaches or disciplines so far apart from each other can constitute a limitation, but also an opportunity. Research on technostress undoubtedly requires greater post-disciplinary collaboration between researchers from both knowledge branches to collaborate closely, complementing each other in addressing holistic studies on the problems and challenges to be faced in the immediate future [99]. This is new when compared to recent bibliometric studies on the use of technology and its effects on mental health, where journals focus mainly on health issues (Psych* and Neuro*) [100].

Definitely, the presence of a relatively small group of articles (147) and researchers (296) on the subject of technostress stands out, which indicates, on the one hand, the topicality, relevance and validity of the subject investigated and, on the other, a line of fairly clear research. A third aspect to consider is the risk of “inbreeding” in the conceptual perspectives used and, in the conclusions, reached above.

Given the need for greater disciplinary cooperation between the psychological and technological approach to technostress, at a second research level a co-authorship analysis was carried out at the country, institution, and author levels. The results obtained show that, at the country level, the United States dominates knowledge production in technostress, but at the institutional level, with a critical research mass, the University of Lancaster is noteworthy and, in particular, the position reached by Tarafdar [5,22,29,101,102,103,104,105,106,107,108,109] among the prolific authors. In addition to dominating research with a technological focus, this English University and its researcher achieve high centrality levels, which allows powerful articulation within the academic network, given the multiple co-authorships that they maintain in a distributed way. In this manner they join six clusters (intra-cohesional), with a dominant presence of techno-scientific pragmatism in only one cluster. Regarding the psychological approach, among the prolific authors Cao and other Chinese and Korean authors stand out, highlighting the performance and centrality in scientific production accomplished by the University of Science and Technology of China [39,110,111,112,113,114]. Thus, the first and second author in the world maintain dissimilar approaches, without presenting joint works and having, as their only link, other researchers from the City University of Hong Kong.

Finally, at the thematic analysis level, the present investigation has revealed that technostress attempts to sustain itself. Although the keywords plus set that nominally identify the articles shows a high connection density of the type “all with all”, other concepts with a recurring presence cannot be identified, but rather the “stress” itself and its obvious “impact”. Despite the above, it is possible to identify correlations between the main keywords plus, which through a systematic review of the literature, in a more specifically detailed way, could establish causality in order to better understand causes, moderators and effects of techno-stress that limit the negative effects of technology. Thematic orientation by common references of the ‘bibliographic coupling’ type also shows a high connection density between the h-index articles, although computationally it is possible to fragment them into two sets of 12 and 17 academic works, although authors can be observed on both sides of the established borderline.

By reviewing in greater detail, the published material on technostress included in this article, it is possible to observe a certain distance between the different theoretical perspectives on stress in the current research on technostress. This distance or lack of communication can be evidenced as follows: (1) of the total of 147 articles reviewed, only 62 include in their theoretical references the perspectives of the authors Selye, Lazarus, Karasek, Siegrest and/or Cooper (see Table 5); (2) the remaining 85 articles opt for a more pragmatic or socio-technical perspective in their approach; (3) reference to authors recognized as referents of the theoretical stress models mentioned in the 62 articles does not necessarily return consistently to the conclusions and/or suggestions of current technostress researchers opting for other paths.

The vision of Lazarus [115] predominates in 48 of the 62 articles that allude to theorists of stress. In all of these, the viewpoint is of a psychological nature, noting evaluation as an individual device to provide both well-being and coping capacity in the face of specifically overwhelming demands. Emotions act as regulating entities of potential overflows, allowing maximum effort with somatopsychic rest periods. Likewise, individual cognitive efforts and the passage to action are perceived as a style of managing specific demands [116].

This is followed in predominance of use by Cooper’s theoretical model mentioned by 36 articles (see Table 6). This approach, although it can be complemented with that of Lazarus [115] in its individual and cognitive dimension, places the emphasis on sources of stress at work, differentiating intrinsic from extrinsic sources. The reviewed articles stress this relationship of sources to various organizational contexts, placing technology as a source of tension that affects the individual and work climate, able to operate both extrinsically and intrinsically, that is, affecting the personality of the organization [46], the personality of the individual and the work-home interface.

Third, and well below the previous number of references, the theoretical model of Karasek [117] appears in eight articles. This model focuses on the job stress perceived by individuals and on their job demand’s control model. Given that demand control involves articulating labor demand and freedom in decision-making, it is foreseeable that it will be used less often. Freedom is restricted by technological devices since, although they can facilitate work, they also restrict it within its technocratic parameters, limiting the domain of the work environment itself.

Fourth, there are five articles that refer to Selye’s proposal [3]. In part, due to the age of this perspective and its initial application with animals, this viewpoint has been discontinued, which is consistent with the decision of most current technostress researchers not to consider it. Those who do consider it, however, re-use it in their contribution to the “general adaptation syndrome” [118], including its three phases: alarm, resistance, and exhaustion.

Lastly, a single article was detected [119] that reflects Siegrist’s proposal [120]. This proposal focuses on the concept of social reciprocity and on the discrepancy between the effort committed by the individual and the material and immaterial reward obtained in her job. This article highlights the role of individual motivation as an intrinsic effort and, in turn, takes up the proposals of Cooper, Karaseck and Lazarus. Siegrest is a follower of Cooper and has outlined both intrinsic and extrinsic compensation levels that allow stress to be coped with, which is considered by Tams et al. [119].

Furthermore, the largest number of articles (85/147) were not published in psychological, clinical, social, or organizational journals, and the training of researchers does not appear to have a psychological basis. In several cases, these are journals attached to categories of Information Science & Library Science or Computer Science, making it highly likely that these articles are aseptic with respect to a psychological approach. They operate via socio-technical approaches, considering factors specific to the socio-labor context, which are extrinsic rather than intrinsic. This tends to make the individual invisible in her attitudes, motivations or personality traits as components of a stressful situation and to focus on the work and the technical devices through which the work assigned in a given job and whether it is efficiently carried out or not. In other words, tension turns towards extra-individual components, understanding the technological as a simile of the stress mediating organism.

Though this study allows a better understanding of this emerging theoretical construct. given its origin in multiple streams of thinking, it does not allow a clear vision of technostress. This information suggests that there is a whole opportunities field available to those researchers who want to contribute post-disciplinary scientific knowledge [99,121,122] to a highly topical subject such as techno-stress, a research area that will surely acquire even more relevance given the mental health alert situation across the planet as a consequence of Covid-19, and that is redesigning the foundations of personal, family, work and social life, with the greater use and penetration of technological tools (even in groups such as elderly adults who have been forced to “be online“ with these technologies to stay in touch with loved ones). These changes, which are permeating our lifestyles, and whose consequences we are not yet aware of, will generate new tensions that will lead to further technostress.

## 6. Conclusions

In conclusion, this paper gives a comprehensive review of technostress research in the academic field. This analysis recognizes leading tendencies in quantity (production), quality (impact), relationship, concentration of prolific authors, nucleus journals, highly cited articles, co-authorship, main themes, and the classic authors used in stress research. In this way, a first meta-analytic, systemic and global approach makes possible a better understanding of the technostress phenomenon and the way in which society and companies can face the dark side of technology, a key issue in the “new normal” scenario, where the hybrid presence and the intensive use of information and communication technologies seem to be more present.

As we have previously said, technostress influences organizational commitment, job satisfaction, and employee outcomes (e.g., absenteeism, turnover) [29]. On the other hand, according to Salanova et al. [30], with chronic anxiety, fatigue, and skepticism users’ ICT self-efficacy is lowered. Regarding the latter issue, the theory of Kumar et al. [123] shows how the negative impact of technostress on job satisfaction and on organizational commitment exists. Therefore, technostress can lead to negative appraisal of one’s job; exactly for this reason, management of technostress depends on how one perceives techno-change and how one interprets it [123]. Since our results indicate that individuals are stressed in these situations, organizations need to provide tools for individuals to deal with information overload, such as technical support; it was also noted that higher levels of task-technology fit lead to lower levels of technostress. Therefore, this could present a useful avenue for organizations to evaluate task and technology context proactively in order to reduce technostress. [124].

## 7. Limitations

This article, given the breadth of the works reviewed at various levels of aggregation, allows us to lay the foundations for expansion of the studies on technostress that will be required in the future. However, given the growth rate of publications, the increase in technological developments that are continually changing the way companies run their businesses, and the short period of time that will elapse until knowledge of technostress ceases to be considered contemporary, a major limitation is precisely its obsolescence, in particular due to the intensified use of information and communication technologies that the global pandemic from Sars-Cov 2 has imposed on us, which could generate changes in this phenomenon, increasing it or creating forms of defense against negative effects which have developed due to excessive use.

ICT is increasingly affecting all aspects of human society, in particular our workplace and daily life, and for this very reason, by maintaining the growth rate of publications on technostress, it is estimated that within five years there may be a turnover total of citable references (with the exception of those that manage to become classics, with high numbers of citations), so at that time this study should no longer have a high level of validity and, for this reason, it would be necessary to go back to studying and systematize the new knowledge generated by that date.

## Figures and Tables

**Table 1:** Type of data, methods and results.

Type of Data	Unit of Analysis	Analytical Methods	Presentations of Results
Publication Year	Article	Exponential regression	Bar and linear graph
Author	Article	Price’s Law	Table
Journal	Article	Bradford’s Law	Table
Citation article	Article	Hirsch index	Box plot
Country, Affiliation, Author, Keywords plus	Article	Co-authorship	Graph
References	Article	Bibliographic coupling	Graph

**Table 2:** Prolific authors.

#	Authors/PhD in	Memberships	Concentration Categories Web of Science (WoS) (%)	Articles
1	Tarafdar M/Management	University of Lancaster, University of Toledo	Information Science & Library Science (75%)	12 ^1^
2	Cao XF/Information Systems, Management Science and Engineering	University of Science and Technology of China, Hefei University of Technology	Psychology (Multidisciplinary; Experimental) (60%)	5
3	Grover V/Management Information Systems	University of Arkansas, Universidad Clemson	Computer Science, Information Systems; Information Science & Library Science (100%)	5
4	Laumer S/Information Systems	Friedrich-Alexander-Universität Erlangen-Nürnberg, Otto-Friedrich-Universität Bamberg	Information Science & Library Science (100%)	5
5	Maier C/Business Informatics and Applied Informatics	Otto-Friedrich-Universität Bamberg	Information Science & Library Science (100%)	5
6	Tu Q/Manufacturing and Technology Management	Rochester Institute of Technology	Computer Science (Cybernetics; Information Systems; Hardware & Architecture; Software Engineering; Theory & Methods) (80%)	5
7	Weitzel T/Information Systems	Otto-Friedrich-Universität Bamberg	Information Science & Library Science (100%)	5
8	Stich JF/Management	Université de Lorraine	Information Science & Library Science (75%)	4
9	Tams S/Management	HEC Montréal	Computer Science, Information Systems; Information Science & Library Science (75%)	4
10	Turel O/Management Information Systems	California State University - Fullerton	Unconcentrated	4
11	Ali A/Management Science and Engineering	University of Science and Technology of China	Psychology (Multidisciplinary; Experimental) (100%)	3
12	Cooper CL/Management Studies	University of Manchester	Information Science & Library Science (67%)	3
13	Luqman A/Business Administration	University of Science and Technology of China	Psychology (Multidisciplinary; Experimental) (100%)	3
14	Masood A/Business Administration	University of Science and Technology of China	Psychology (Multidisciplinary; Experimental) (100%)	3
15	Ragu-Nathan TS/Management Information Systems	University of Toledo	Computer Science (Information Systems; Hardware & Architecture; Software Engineering; Theory & Methods) (100%)	3
16	Shu Q/n.d.	Xi’an Jiaotong University	Computer Science (Cybernetics; Hardware & Architecture; Software Engineering; Theory & Methods) (67%)	3
17	Stacey P/Management	Loughborough University	Information Science & Library Science (67%)	3
18	Wang KL/Management Science & Engineering	Renmin University of China, Xi’an Jiaotong University	Computer Science (Cybernetics; Hardware & Architecture; Software Engineering; Theory & Methods) (67%)	3
19	Yu LL/Business Administration	University of Science and Technology of China	Information Science & Library Science (67%)	3

^1^ Contributions as sole author or co-author.

**Table 3:** Bradford zones.

Zone	Number Articles (%)		Journals (%)		Bradford Multipliers
1	51	(35%)	7	(9%)	
2	46	(31%)	25	(30%)	3.6
3	50	(34%)	50	(61%)	2.0
Total	147		82		2.8

**Table 4:** Sources in the Bradford core.

#	Source Titles	WoS Categories	JCR Impact Factor	Quartile	Records	% of 147
1	Computers in Human Behavior	Psychology, Multidisciplinary; Psychology, Experimental	4.306	Q1	23	16%
2	Journal of Management Information Systems	Computer Science, Information Systems; Information Science & Library Science; Management	3.013	Q1	6	4%
3	Information Systems Journal	Information Science & Library Science	3.286	Q1	5	3%
4	Journal of The Association for Information Systems	Computer Science, Information Systems; Information Science & Library Science	3.103	Q1	5	3%
5	Behaviour & Information Technology	Computer Science, Cybernetics; Ergonomics	1.429	Q3	4	3%
6	Information Technology & People	Information Science & Library Science	1.263	Q3	4	3%
7	Telematics and Informatics	Information Science & Library Science	3.714	Q1	4	3%

Highlights the journal ‘Computers in Human Behavior’ in zone 1 given its high publication concentration that exceeds 15%.

**Table 5:** Reference articles in the bibliographic coupling.

Cluster	Items	Articles with Common References, First Author (Year) ^3^
Cluster 1 (red)	17	Al-Fudail (2008), Ayyagari (2011), Brooks (2015), Bucher (2013), Day (2012), Galluch (2015), Hudiburg (1989), Lee (2014), Riedl (2012), Salanova (2013), Scott (2005), Shu (2011), Tarafdar (2007), Tarafdar (2010), Tarafdar (2011), Tu (2005), Wang (2008).
Cluster 2 (green)	12	D’arcy (2014), Elie-Dit-Cosaque (2011), Fuglseth (2014), Lee (2016), Luqman (2017), Maier (2015a), Maier (2015b), Ravindran (2014), Reinecke (2017), Tarafdar (2013), Yun (2012), Zhang (2016).

^3^ First author and year indicated only. Complete reference list in Appendix B (Table A1).

**Table 6:** Classic author presence in the technostress articles analyzed.

Classic Author	Used as a Reference in Articles ^4^:
Lazarus	48
Cooper	36
Karasek	8
Selye	5
Siegrest	1

^4^ Complete reference list in Appendix B (Table A2).

## Appendix A

In this appendix the Bradford zone calculation is reported, as indicated in Table 2. Given, a core zone a = 7 and a mean multiplier n = 2.8, the resulting geometric series summation (S_SB_) is:

(1) $$S_{SB}=\overset{3}{\underset{i=1}{\sum }}\left(a*n^{i-1}\right)\;=7+20+54=81$$

with an error percentual margin (ε_p_) of:

(2) $$\varepsilon_{p\;=\;\left(\frac{\left(Real-Estimated\right)}{Real}\right)\;*\;100\;=\;\left(\frac{\left(82-81\right)}{82}\right)\;*\;100\;=\;1.4\%}$$

Error that is considered not significant [76] (p. 228), recognizing as a Bradford nucleus the set of seven journals indicated in Table 3, for the subject and period studied.

## Appendix B

The complete reference list identified as bibliographic coupling in Table 5 is presented in this appendix.

**Table A1:** Complete reference list identified as bibliographic coupling.

Cluster	Articles:	Digital Object Identifier (doi):	Reference:
Cluster 1:	Al-Fudail (2008)	10.1016/j.compedu.2007.11.004	[125]
	Ayyagari (2011)	not available	[20]
	Brooks (2015)	10.1016/j.chb.2014.12.053	[37]
	Bucher (2013)	10.1080/1369118X.2012.710245	[38]
	Day (2012)	10.1037/a0029837	[126]
	Galluch (2015)	not available	[127]
	Hudiburg (1989)	10.2466/pr0.1989.64.3.767	[128]
	Lee (2014)	10.1016/j.chb.2013.10.047	[90]
	Riedl (2012)	10.1007/s12599-012-0207-7	[129]
	Salanova (2013)	10.1080/00207594.2012.680460	[4]
	Scott (2005)	10.1177/0093650205281054	[130]
	Shu (2011)	10.1080/10447318.2011.555313	[131]
	Tarafdar (2007)	10.2753/MIS0742-1222240109	[22]
	Tarafdar (2010)	10.2753/MIS0742-1222270311	[99]
	Tarafdar (2011)	10.1145/1995376.1995403	[29]
	Tu (2005)	10.1145/1053291.1053323	[132]
	Wang (2008)	10.1016/j.chb.2008.05.007	[133]
Cluster 2:	D’arcy (2014)	10.2753/MIS0742-1222310210	[134]
	Elie-Dit-Cosaque (2011)	10.2753/MIS0742-1222280306	[135]
	Fuglseth (2014)	10.1016/j.chb.2014.07.040	[136]
	Lee (2016)	10.1016/j.chb.2015.08.011	[137]
	Luqman (2017)	10.1016/j.chb.2017.01.020	[108]
	Maier (2015a)	10.1057/ejis.2014.3	[138]
	Maier (2015b)	10.1111/isj.12068	[139]
	Ravindran (2014)	10.1002/asi.23122	[140]
	Reinecke (2017)	10.1080/15213269.2015.1121832	[141]
	Tarafdar (2013)	10.1111/isj.12015	[5]
	Yun (2012)	10.2753/JEC1086-4415160405	[142]
	Zhang (2016)	10.1016/j.im.2016.03.006	[143]

The complete reference list identified by relevant authors in Table 6 is presented in this appendix.

**Table A2:** Complete reference list.

Classic Author	Used as a Reference in ^5^:	Total Articles
Lazarus	Adam, 2017; Al-Ansari, 2019; Al-Fudail, 2008; Ayyagari, 2011; Bucher, 2013; Cao, 2018; Chen, 2019; Choi, 2016; D’Arcy, 2014; Day, 2012; de Guinea, 2016; Dong, 2020; Elie-Dit-Cosaque, 2011; Galluch, 2015; Gaudioso, 2017; Goetz, 2020; Hauk, 2019; Hudiburg, 1989; Joo, 2016; Khedhaouria, 2019; Lee, 2016; Lee, 2016b; Lee, 2016c; Loiacono, 2018; Luqman, 2017; Ma, 2019; Maier, 2015; Maier, 2019; Pirkkalainen, 2019; Qi, 2019; Reinecke, 2017; Riedl, 2012; Salo, 2019; Shu, 2011; Stich, 2017; Stich, 2019; Stich, 2019b; Tams, 2014; Tams, 2018; Tams, 2018b; Tarafdar, 2010; Tarafdar, 2011; Tarafdar, 2019; Wang, 2008; Wang, 2019; Yin, 2018; Yu, 2018; Zhang, 2019.	48
Cooper	Al-Ansari, 2019; Al-Fudail, 2008; Ayyagari, 2011; Cao, 2019; Choi, 2016; Day, 2012; Fuglseth, 2014; Galluch, 2015; Gaudioso, 2017; Han, 2018; Khuntia, 2015; Kim, 2015; Lee, 2016; Lee, 2016b; Lee, 2016c; Luqman, 2020; Maier, 2015; Maier, 2019; Qi, 2019; Salanova, 2013; Salo, 2019; Steelman, 2017; Stich, 2017; Stich, 2019; Stich, 2019; Suh, 2017; Tams, 2014; Tams, 2018; Tams, 2018b; Tarafdar, 2007; Tarafdar, 2010; Tarafdar, 2019; Wang, 2020; Yan, 2013; Yu, 2018; Zheng, 2016.	36
Karasek	Elie-Dit-Cosaque, 2011; Galluch, 2015; Lee, 2016; Lee, 2016b; Ma, 2019; Richardson, 2017; Stich, 2019; Tams, 2018	8
Selye	Chen, 2019; Galluch, 2015; Riedl, 2012; Stich, 2019; Tarafdar, 2019.	5
Siegrest	Tams, 2018.	1

^5^ First author and year only are indicated.
